# Uterine Rescue in High-Risk Gestational Trophoblastic Neoplasia Treated with EMA-CO by Uterine Arteries Embolization due to Arteriovenous Malformations

**DOI:** 10.1055/s-0041-1725054

**Published:** 2021-05-12

**Authors:** Arlley Cleverson Belo da Silva, Jurandir Piassi Passos, Roney Cesar Signorini Filho, Antonio Braga, Rosiane Mattar, Sue Yazaki Sun

**Affiliations:** 1 Department of Obstetrics, Escola Paulista de Medicina, Universidade Federal de São Paulo, São Paulo, SP, Brazil; 2 Maternity School, Universidade Federal do Rio de Janeiro, Rio de Janeiro, RJ, Brazil

**Keywords:** gestational trophoblastic disease, gestational trophoblastic neoplasia, EMA-CO protocol, uterine artery embolization, high-risk pregnancy, doença trofoblástica gestacional, neoplasia trafoblástica gestacional, protocolo EMA-CO, embolização de artéria uterina, gravidez de alto risco

## Abstract

Complete hydatidiform mole (CHM) is a rare type of pregnancy, in which 15 to 20% of the cases may develop into gestational trophoblastic neoplasia (GTN). The diagnostic of GTN must be done as early as possible through weekly surveillance of serum hCG after uterine evacuation. We report the case of 23-year-old primigravida, with CHM but without surveillance of hCG after uterine evacuation. Two months later, the patient presented to the emergency with vaginal bleeding and was referred to the Centro de Doenças Trofoblásticas do Hospital São Paulo. She was diagnosed with high risk GTN stage/score III:7 as per The International Federation of Gynecology and Obstetrics/World Health Organization (FIGO/WHO). The sonographic examination revealed enlarged uterus with a heterogeneous mass constituted of multiple large vessels invading and causing disarrangement of the myometrium. The patient evolved with progressive worsening of vaginal bleeding after chemotherapy with etoposide, methotrexate, actinomycin D, cyclophosphamide and vincristine (EMA-CO) regimen. She underwent blood transfusion and embolization of uterine arteries due to severe vaginal hemorrhage episodes, with complete control of bleeding. The hCG reached a negative value after the third cycle, and there was a complete regression of the anomalous vascularization of the uterus as well as full recovery of the uterine anatomy. The treatment in a reference center was essential for the appropriate management, especially regarding the uterine arteries embolization trough percutaneous femoral artery puncture, which was crucial to avoid the hysterectomy and allow GTN cure and maintenance of reproductive life.

## Resumo

Mola hidatiforme completa (MHC) é um tipo raro de gravidez, na qual 15 a 20% dos casos podem desenvolver neoplasia trofoblástica gestacional (NTG). O diagnóstico de NTG deve ser feito o mais cedo possível, pelo monitoramento semanal do hCG sérico após esvaziamento uterino. Relatamos o caso de uma paciente primigesta, de 23 anos de idade, com MHC, sem vigilância de hCG após esvaziamento uterino. Dois meses depois, a paciente compareceu na emergência com sangramento vaginal, sendo encaminhada ao Centro de Doenças Trofoblásticas do Hospital São Paulo, onde foi diagnosticada com NTG de alto risco, estádio e score de risco III:7 de acordo com a *The International Federation of Gynecology and Obstetrics* /Organização Mundial de Saúde (FIGO/OMS). O exame ultrassonográfico revelou útero aumentado com uma massa heterogênea constituída por múltiplos vasos volumosos invadindo e desestruturando o miométrio. A paciente evoluiu com piora progressiva do sangramento vaginal após quimioterapia com o regime *etoposide, methotrexate, actinomycin D, cyclophosphamide and vincristine* (EMA-CO). Ela foi submetida a transfusão de sangue e embolização das artérias uterinas devido aos episódios graves de hemorragia vaginal, com completo controle do sangramento. O hCG atingiu valor negativo após o terceiro ciclo, havendo regressão completa da vascularização uterina anômala, assim como recuperação da anatomia uterina. O tratamento em um centro de referência permitiu o manejo adequado, principalmente no que se refere à embolização das artérias uterinas através da punção percutânea da artéria femoral, que foi crucial para evitar a histerectomia, permitindo a cura da NTG e a manutenção da vida reprodutiva.

## Introduction

Gestational trophoblastic disease (GTD) is a disorder of pregnancy caused by defective differentiation of the trophoblast with both benign and malignant spectrum. As premalignant forms, there are the complete (CHM) and partial hydatidiform mole (PHM). The malignant forms are known as gestational trophoblastic neoplasia (GTN) and classified into invasive mole, choriocarcinoma, placental trophoblastic tumor and epithelioid trophoblastic tumor. 1 Diagnosis of GTN is performed based on the criteria of the International Federation of Gynecology and Obstetrics (FIGO) published first in 2002 2 and updated in 2018. 3 The updated criteria consist in rising or stabilization of the serum level of βHCG over at least a period of two or three weeks, respectively, or the histologic diagnosis of choriocarcinoma. 3

Gestational trophoblastic disease among women with reproductive desire is treated with chemotherapy, with high chance of cure even in advanced stages. 3 However, embolization of the uterine arteries may represent an alternative approach to avoid hysterectomy due to massive bleeding from uterine arteriovenous malformation owing to myometrial tumoral infiltration. 4 5

The present case report was approved by the Ethics Review Board of Universidade Federal de São Paulo, under the number 4.099.490. The need for informed consent was waived due to unsuccessful attempts to contact the patient by email and phone number registered on the service database and patient record.

## Case Report

**Fig. 1:**
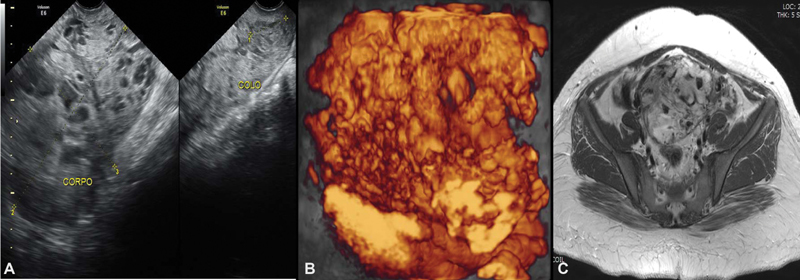
Uterine images before chemotherapy (June 9 ^th^ , 2016) demonstrating loss of myometrium stratification due to heterogeneous image, mainly vascular, invading the whole uterus and cervix. A. Pelvic transvaginal ultrasonography B-mode. B. Transvaginal three-dimensional HD-flow multiplanar view scan. C. Pelvic magnetic resonance imaging (MRI)

**Fig. 2:**
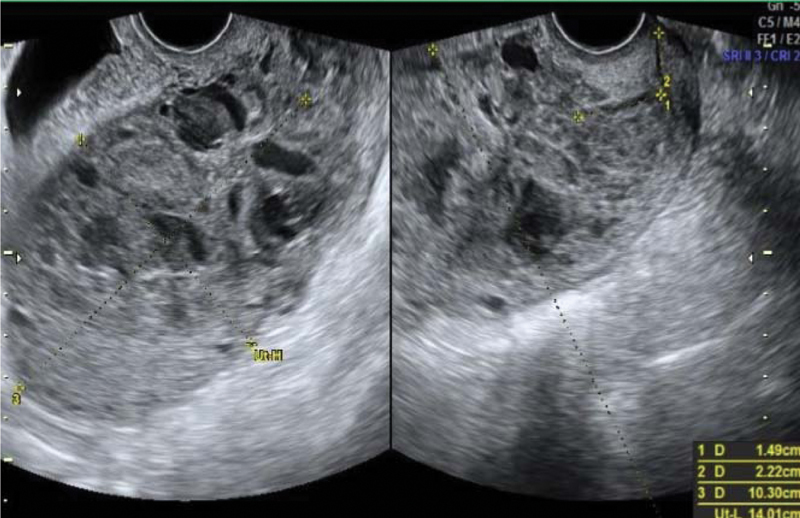
Transvaginal scan before the second cycle of polychemotherapy (June 25 ^th^ , 2016)

**Fig. 3:**
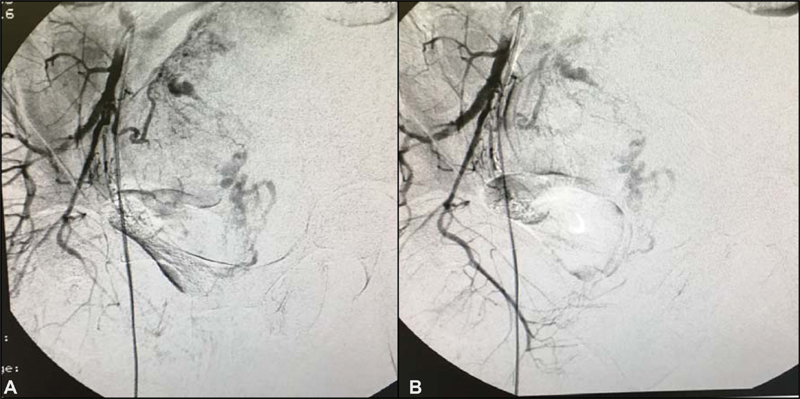
Pelvic angiogram. A. Ongoing embolization. B. Completed embolization (July 6 ^th^ , 2016)

**Fig. 4:**
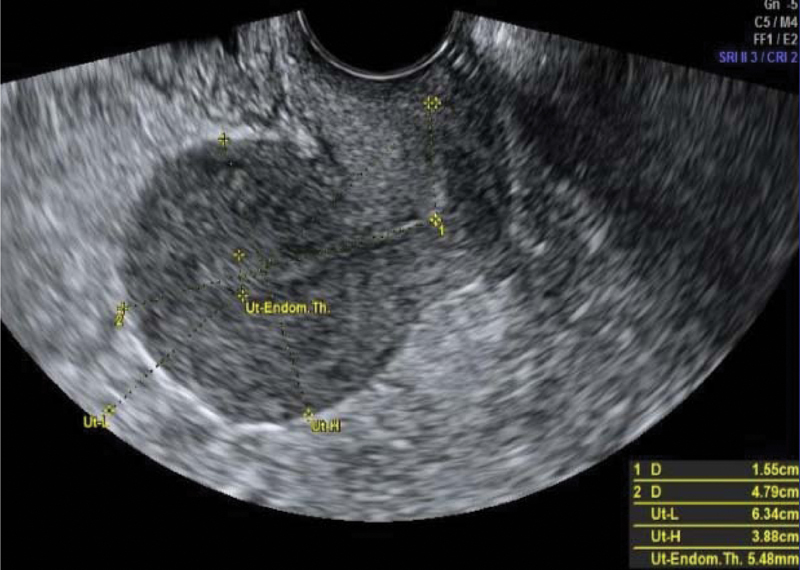
Transvaginal scan after the last cycle of polychemotherapy (December 14 ^th^ , 2016)

Our patient was a 23-year-old primigravida. She presented to the emergency room with vaginal bleeding at 18 weeks of gestation and, after an investigative transvaginal scan, she was diagnosed with CHM. The patient underwent uterine evacuation by manual intrauterine aspiration. The first βhCG measurement was ˃ 225,000 mlU/ml. Other blood test results showed hemoglobin 9.7 g/dL; hematocrit 30%; TSH 0.003 ml/mL; and free T4 1.69 ng/dL. The result of the histology confirmed the CHM diagnosis. The patient had missed surveillance of hCG for 2 consecutive months after the uterine evacuation. After this period, she went to the emergency room with vaginal bleeding and was subsequently referred to Centro de Doenças Trofoblásticas do Hospital São Paulo, where she was diagnosed with gestational trophoblastic neoplasia (GTN) III:7. The sonographic examination revealed an enlarged uterus measuring 607.8 cm ^3^ with a heterogeneous mass measuring 10.0 × 15.9 × 7.3 cm, constituted of multiple large vessels invading and causing disarrangement of the myometrium ( Figs. 1 and 2 ). Polychemotherapy with etoposide, methotrexate, actinomycin D, cyclophosphamide and vincristine (EMA-CO) regimen was immediately prescribed. Since her admission, the patient had had vaginal bleeding that progressively worsened after starting polychemotherapy, probably due to rupture of vessels visualized previously. She received four red blood cells units' transfusion during the second cycle of EMA-CO, and due to the persistence of the severe vaginal hemorrhage, embolization of the uterine arteries was performed successfully as a treatment. Embolization was performed by percutaneous puncture accessing the right femoral artery, and each uterine artery was selectively catheterized with a 5-F glide catheter and embolized with geolfoam particles ( Fig. 3 ). Although the patient had shown liver toxicity after the first cycle of chemotherapy, the treatment could be done without delay after adjusting the doses. The hCG reached a negative value ( Table 1 ) after the third cycle, and there was a complete regression of anomalous vascularization of the uterus as well as recovery of the uterine anatomy seen by pelvic ultrasonography ( Fig. 4 ). The patient was followed monthly until September 2017 and, later, every 3 months up to July 2018 under hCG surveillance and contraception recommendation.

**Table 1:** Clinical and laboratorial data

Date	Event	hCG mUI/mL	Others
April 14 ^th^ , 2016	Molar suction uterine evacuation	> 22,5000	Hb 9.7 g/dL, Htc 30%
June 9 ^th^ , 2016	Referred to CDTHSP	229,965	
June 10 ^th^ and 11 ^th^ , 2016	EMA		
June 17 ^th^ , 2016	CO		
June 28 ^th^ and 29 ^th^ , 2016	EMA	24,633	Hb = 10.1 g/dL; Htc 29.3%
July 5 ^th^ , 2016	CO		Hb = 6.5 g/dL, Htc = 20.3% Transfusion 4 RBC SBP = 90 × 60 mm Hg, CF = 120 bpm
July 6 ^th^ , 2016	Embolization of the uterine arteries	−	
July 12 ^th^ and 13 ^th^ , 2016	EMA	1,137	
July 25 ^th^ 2016	CO		
August 2 ^nd^ and 3 ^rd^ , 2016	EMA	4.5	
August 9 ^th^ , 2016	CO		
August 16 ^th^ and 17 ^th^ , 2016	EMA	3	
August 23 ^rd^ , 2016	CO		
August 30 ^th^ and 31 ^st^ , 2016	EMA	1.3	
September 6 ^th^ , 2016	CO		

Abbreviations: CDTHSP, Centro de Doenças Trofoblásticas do Hospital São Paulo; CF, cardiac frequency; EMA-CO chemotherapy regimen: etoposide, methotrexate, actinomycin D, cyclophosfamide, vincristine; Hb, hemoglobin; hCG, human chorionic gonadotropin; htc, hematocrit; RBC, red blood cells; SBP, systolic blood pressure.

## Discussion

Vaginal bleeding has been reported as the most common symptom in the cases of hydatidiform mole. However, the symptoms and signs that have been associated with molar pregnancy are getting less common in the practice due to the greater availability of ultrasound scans and hCG tests in the first trimester of pregnancy, as a routine or to evaluate vaginal bleeding, affording the earlier diagnosis of molar pregnancy. 6 7 In the present case report, the patient sought the emergency room due to vaginal bleeding, but without other symptoms such as hyperemesis, respiratory discomfort, and hyperthyroidism, which could indicate a more advanced disease.

In view of the initial hydatidiform mole diagnosis, the management was accomplished by performing a uterine evacuation in the operating room, as recommended. We chose the suction evacuation technique of manual vacuum aspiration, which has been associated with lower risk of uterine synechia, compared with electric vacuum aspiration. 8

The diagnosis of GTN is based on surveillance of serum levels of hCG, and this malignant process is suggested by the presence of plateau or rising of hCG serum levels on two or three consecutive weekly samples. 3 9 Our patient had not performed the recommended surveillance of serum hCG levels after the uterine evacuation; then, 2 months later, she evolved with an elevation of hCG serum levels associated with vaginal bleeding. These features along with the pelvic ultrasonography demonstrating lesions on the uterine wall allowed the diagnosis of GTN.

Once the diagnosis of GTN has been made, the anatomical involvement of the disease and risk should be defined based on the FIGO criteria ( Charts 1 2 3 ), and the patient must be classified as low or high risk. Patients with a score of 0 to 6 are defined as low risk because they are likely to respond to single-drug therapy, and those with a score higher than 6 are considered as high risk of resistance to single-drug chemotherapy. According to this classification, the multiple drugs chemotherapeutic regimen is preferred for the high-risk patients. 1 3

**Chart 1:** Gestational trophoblastic neoplasia anatomic staging (FIGO/WHO)

Stage I	Disease confined to the uterus
Stage II	GTN extends outside of the uterus but limited to the genital structures (adnexa, vagina, broad ligament)
Stage III	GTN extends to the lungs, with or without know genital tract involvement
Stage IV	All other metastatic sites

Abbreviations: FIGO/WHO, The International Federation of Gynecology and Obstetrics/World Health Organization; GTN, gestational trophoblastic neoplasia.

**Chart 2:** Gestational trophoblastic neoplasia prognostic scoring system (FIGO/WHO)

Scores	0	1	2	4
Age	< 40	≥ 40	−	−
Antecedent pregnancy	Mole	Abortion	Term	−
Interval months from index pregnancy	< 4	4–7	7–13	≥ 13
Pretreatment serum hCG (IU/Ml)	< 10 ^3^	10 ^3^ –10 ^4^	10 ^4^ –10 ^5^	≥ 10 ^5^
Largest tumor size (including uterus)	−	3–5 cm	≥ 5 cm	−
Site of metastasis	Lung	Spleen, kidney	Gastrointestinal	Liver, brain
Number of metastasis	−	1–4	5–8	> 8
Previous failed chemotherapy	−	−	Single drug	2 or more drugs

Abbreviations: FIGO/WHO, The International Federation of Gynecology and Obstetrics/World Health Organization; hCG, human chorionic gonadotropin.

After the confirmed diagnosis of GTN, a further investigation is required, and taking into consideration that pulmonary metastases are the most common ones, a chest radiograph is crucial. If lesions are noted on chest X-ray, magnetic resonance imaging (MRI) of the brain and abdominal computed tomography (CT) are indicated to exclude metastatic disease in other sites, such as the liver. 2 3 6 In the present case, the patient was classified as high risk and had pulmonary metastasis identified. In view of the findings, a multiple agent therapy was indicated, and the regimen with EMA-CO was chosen for being considered in the literature the first-line therapy for high-risk cases. 1 3 6 It is essential to highlight the importance of rigorous postmolar follow-up for early detection of GTN, which would enable less aggressive regimens of treatment, such as in this case. 10

The toxicity of the EMA-CO regimen is usually well tolerated, being more common the hematological; however, there are also reports of effects on the gastrointestinal tract and peritoneal and pleural serositis. 11 The patient in the current case showed toxicity in the gastrointestinal tract with the use of the EMA-CO chemotherapy regimen, which could be managed through an adjustment of the doses.

The large vessels of the arteriovenous malformations invading the myometrium in communication with the endometrial cavity, almost reaching the serosa, represented a threat of vaginal bleeding and uterine perforation. Embolization of uterine arteries was an alternative approach to control the vaginal bleeding and avoid hysterectomy in this nulliparous patient.

Studies in the literature support a high pregnancy rate despite prior chemotherapy with no increased adverse outcomes. 1 9 12 Furthermore, as previously demonstrated, women of childbearing age were able to get pregnant after uterine arteries embolization, 13 14 15 this management was adopted when the patient was found having severe hemorrhage episodes over the chemotherapy.

In addition, a previous study showed that patients who underwent uterine artery embolization to treat hemorrhage from arteriovenous malformation due GTN had favorable outcomes with low rate of complications and recovered well after the procedure, with normal uterine function and regular menstrual cycles. 16

Owing to the complexity of the disease and its severity, patients must be treated and followed-up in a specialized center with structure and experience in the management of gestational trophoblastic disease, therefore increasing survival as well as the chances of cure of patients with this disease. 4

## Conclusion

In the case presently reported, the fact that the patient was referred to a tertiary and specialized service in GTD was extremely important for the successful outcome. In these reference centers, a multidisciplinary team is available to offer the best treatment to cure the patient and maintain her reproductive function. Selective embolization of uterine arteries is one of the most effective techniques to avoid hysterectomy due to intractable pelvic hemorrhage in cases of GTN in nulliparous women.
